# The Lymphatic System

**Published:** 1915-11

**Authors:** 


### The Lymphatic System.

While the editor has felt that he should not publish strictly medical articles dealing with his own experience in this journal, but should submit them to the impartial judgment of other editors, it does not seem that the editorial department should either ignore practical medical problems nor that a general reference to personal experience is out of place.

In the last few years, our attention has been called to the lymphatic system by a case of mesenteric cyst of the chylous type, and by two other cases in which this same form of mesenteric cyst was considered probable; also by two cases of obstruction to the lymphatic circulation and by another seen in a hospital abroad. Cases of chylous accumulation in body cavities have also been reported, including one or two from this vicinity.

In studying these cases and reports, the paucity of literature has been impressed upon us. The lymphatic system is discussed in works on anatomy, physiology and physiologic chemistry, but only in a perfunctory way in works on the practice of medicine, surgery or even pathology. As to therapeutics, we have not only been forced to admit personal incapacity, but have been handicapped by an almost total lack of available general information. Two centuries ago this was not the case. The contemporaneous discovery of the lymphatic circulation, or perhaps one should say the discovery of its significance, was prominent in the mind of the profession and the lymphatic system was considered, not merely as part of scientific knowledge but as a field for the clinician. Unfortunately, the doctors of this time "knew many things that were not so," especially in their frequent and, in the light of modern discoveries, obvious confusion of pus with chyle. Nevertheless, a perusal of early literature is far from valueless.

While lesions and disturbances of the lymphatic system are rare, or, at least, are rarely recognized, beyond the participation of the lymphatics in septic and malignant processes, they do occur, perhaps more frequently than is realized. For example, it is possible that many cases of dropsy, assigned to the kidneys, blood or blood-vascular system, are essentially due to lesions of the lymphatic channels or the receptaculum. We would, therefore, call attention to the lymphatics as a field of study for practicing physicians and would welcome articles dealing with the subject.

